# Risk Behaviors for Reproductive Tract Infection in Women Who Have Sex with Women in Beijing, China

**DOI:** 10.1371/journal.pone.0040114

**Published:** 2012-07-02

**Authors:** Xiaofang Wang, Jessie L. Norris, Yingjie Liu, Sten H. Vermund, Han-Zhu Qian, Ling Han, Ning Wang

**Affiliations:** 1 National Center for AIDS/STD Control and Prevention, Chinese Center for Disease Control and Prevention, Changping District, Beijing, People's Republic of China; 2 Institute for AIDS/STD Control and Prevention, Beijing Chaoyang District Center for Disease Control and Prevention, Chaoyang District, Beijing, People's Republic of China; 3 Vanderbilt Institute for Global Health, Departments of Pediatrics and Medicine, Vanderbilt University School of Medicine, Nashville, Tennessee, United States of America; 4 Department of Sociology, University of California San Diego, San Diego, California, United States of America; UCL Institute of Child Health, University College London, United Kingdom

## Abstract

**Objectives:**

To assess risk behaviors for reproductive tract infections (RTI) including sexually transmitted infections (STI) among women who have sex with women (WSW) in Beijing, China.

**Methods:**

A cross-sectional study of women recruited from venues and internet outreach analyzed using interviews.

**Results:**

We recruited 224 WSW, among whom were 37 couples. The average age of participants was 25.6 years. Sex with men in the past year was reported by 10.7% of participants. During the past year, 34.3% (77/224) had had >1 sexual partner and 72.4% (162/224) had ever had >1 sexual partner. Condom use in the last sex with a man was reported by 54.2% (13/24) of women; 12.5% (3/24) reported never having used a condom with a man in the past year. In the past year, 13.4% (30/224) reported using sex toys with their female partners; of these, 43.3% (13/30) reported consistent condom use with the sex toys and 36.7% (11/30) had shared sex toys. Among participants 65.2% (120/184) reported that their “G-spot” had been stimulated during sex, 49.2% (59/120) of whom reported bleeding during or after sex. Only 12.5% (8/64) of those never reporting “G spot” stimulation reported bleeding during or after sex (P<0.001).

**Conclusions:**

WSW in Beijing engaged in high-risk sexual behaviors that may carry a substantial risk of being infected with STI/RTI. To implement STI/RTI prevention and intervention among women, women-women sexual behavior should be considered when doing research and intervention programs.

## Introduction

In China, sexually transmitted infection (STI)/human immunodeficiency virus (HIV)-related interventions typically engage men who have sex with men (MSM). We are aware of none, however, that have targeted women who have sex with women (WSW). Policymakers in China may believe that WSW have no risk of STI/HIV infection because traditional Chinese culture does not even recognize that women engage in same-sex behavior. Some non-Chinese studies suggest that the risk of transmitting STI/HIV between women is low [1], [2], [3], [4], [5]. Some argue that this low STI risk for WSW excuses their less frequent Pap smears [6], [7], [8]. In fact, sexual contact between women has been associated with transmission of STIs and reproductive tract infections (RTIs) such as human papillomavirus [9], Herpes simplex virus type 2 (HSV2) [10], [11], bacterial vaginosis (BV) [12], candidiasis [13], syphilis [14], and other STIs [6], [15], [16], [17], [18]. Prior research about WSW sexual health in other countries indicates that up to 44% of WSW have a lifetime history of ≥1 STI.

WSW may be engaging in behaviors that increase their risk of STI infection. STI transmission among WSW has been documented [19], [20], even among WSW who had no previous sexual contact with men [21], most likely via digital-vaginal contact and/or use of shared sex toys [2], [6], [9], [12], [15], [16], [17], [19], [22], [23], [24], [25], [26]. As a subpopulation of WSW, women who have sex with both men and women may also have a true level of risk that is underreported. Their STI/HIV risk may actually exceed that of women who only have sex with men due to associated behavioral factors such as high-risk sex with men, drug use, and commercial sex work [10], [18], [27], [28], [29], [30].

It is difficult to determine the total number of Chinese WSW who may be at risk of STI infection. The WSW label categorizes women according to a behavioral act rather than a sexual orientation. Thus, WSW includes self-identified lesbians and bisexuals as well as heterosexual women who engage in homosexual behaviors. In the U.S. National Health and Nutrition Examination Surveys 2001–2006, women-women sex was reported by 7.1% of women aged 18 to 59 [11]. A national Chinese survey of college students in 2001 showed that the women-women sexual contact rate among 3,023,004 female college students was 16.8%, while 4.6% of all female respondents self-identified as having same-sex orientation [31]. Researchers have hypothesized that the sizes of the WSW population in many countries have been underestimated [3], [32]. While women outside college campuses may have lower rates of same-sex sexual behavior, WSW would still comprise a large population in China. Based on our formative research among WSW in Beijing, we studied the extent to which sexually active Chinese WSW are engaging in behaviors that have been previously identified as putting them at risk of RTIs, including STIs and HIV.

## Methods

### Recruitment, study site, and population

We recruited 224 WSW aged 18 years old or over by means of convenience sampling from September 2010–April 2011 in Beijing, China. For the purposes of this study, the category “WSW” was defined to encompass both women who only have sex with women, and women who have sex with women and men (WSWM). Recruitment was announced over the internet through lesbian activist groups, LGBT (lesbian, gay, bisexual, transgender) websites, lesbian forums, “QQ” chatroom groups, and well-known lesbian and social activist blogs. QQ is the most widely used chatroom software in China; in 2011 the current user pool surpassed 140 million persons [33]. During the study, timely messages were disseminated to participants through appropriate public or private means on such topics as the number of volunteers, the clinical results of volunteers, and volunteer feedback to the investigators. A personal blog was established (by XW) to educate participants on key sexual health concepts, based on commonly expressed misconceptions. The personal blog provided female sexual health tips, discussed common health problems among survey participants and gynecologists' suggested treatments; and offered personal reflections on the recruiting process and the study itself as a personal touch that helped participants bond with the principal investigator (XW). The blog link and study contact information (QQ number, email address and cell-phone number), were included in the study's publicity materials. To avoid bias, the blog did not publish information from the survey questions or their results until the end of the study, though basic sexual health information was provided throughout.

To promote participation, the PI (XW) reviewed the study and distributed leaflets during WSW-focused sexual health presentations in Beijing-area WSW venues, which included two bars, a salon, and two non-governmental organization sites. In both video and radio programs, the research and related lesbian sexual health issues were discussed through internet resources focused on lesbians. For the video program, a lesbian couple was invited to introduce the study; in the radio program, the PI discussed the study in an interview with the host. Recruiting measures were designed to establish trust within the lesbian community, encouraging WSW to participate in the survey and to speak frankly about stigmatized behaviors. Participants were encouraged to refer eligible friends and their female partners.

During the study, participants were invited to an “LGBT center” (lesbian, gay, bisexual, transgender) in the first month and a hospital in the following seven months in Beijing between 8am and 4pm on weekends. The location was changed to a hospital after the first month because its accessibility to the target population. The participants read and signed a written informed consent statement before being enrolled in the study. Sex between women determined eligibility and was defined as genital contact including oral, vaginal or anal sex. Of 236 women who came to the survey location, three had had no sex with another woman and one was transgender (physically a male but self-identifying as a lesbian), and were excluded. Seven women were surveyed but had to postpone the gynecological exam until their second meeting because they were menstruating the first time they came. One woman withdrew due to dizziness during the blood draw. Hence, 224 of the 229 unique women (98%) who came to enrollment were included in the survey.

### Survey and interview

The survey was completed through a face to face interview with one of four trained interviewers. Each participant was provided an 8-digit identification number and asked to respond to a questionnaire that recorded their demographic information and sexual background and experience, including risk factors for STI/HIV transmission [18], [20], [28], [29], [30]. Volunteers were also asked on the survey about their first sexual experience, defined in this study as “first genital contact, including receiving oral, vaginal or anal sex.” Each volunteer received a 30RMB ($4.69 US) mobile phone credit and two finger condoms.

We designed the survey based on instruments used in previous studies of WSW [6], [34], [35], [36], [37], [38] and Chinese men who have sex with men (MSM) [39], [40], as well as insights from both key informant and focus group discussions with WSW. The PI (XW) reviewed a complete version of the MSM survey and received assistance from the MSM survey's authors on designing the survey for this study. Internal validation data for the MSM survey was not reported. A draft questionnaire for this study was piloted with 11 WSW before being finalized.

Contents of the survey, which participants completed anonymously, included demographic information (e.g., marital status, Beijing residency, education, profession, income) and sexual history (e.g., sexual orientation, age of sexual debut, gender of first sexual partner, age of woman-woman sex debut, specific sexual behaviors, number of sexual partners, G-spot exploration, commercial sex, and heterosexual intercourse in the past year).

In China, each household has a residence card registered to a specific location, to control intercity migration. Participants were asked whether their hukou (household residence card) was registered to Beijing or to another location. Participants were defined as having “Beijing local residency” if they currently had a Beijing hukou and had been born in Beijing. This was used to estimate the percentage of participants who originally came from outside Beijing.

Participants were also asked on the survey about STI risk factors such as alcohol consumption and drug use. Participants were asked if they drank alcohol, how often, in what quantities, at which age they first consumed alcohol and if they had used alcohol during sexual activity in the last year. Participants were also asked if they had ever used drugs, which drugs (heroin, ecstasy, methamphetamine, ketamine, or ‘other’) they had used, if they had used drugs before or after sexual activity, if they had used drugs in the last year, and whether and how often they had shared needles.

Participants were also asked if they were aware of the “G-spot” and if they or their partners deliberately sought it during sexual activity. The G-spot or Grafenberg spot was firstly coined by Addiego et al in 1981 to recognize Dr. Grafenberg, who first reported the existence of the area in 1950 [41], [42]. The G-spot in this study refers to a small but highly sensitive area on the anterior wall of the vagina, about a third of the way up from the vaginal opening.

### Data statistics and analysis

EpiData software (EpiData 3.0 for windows, The EpiData Association, Odense, Denmark) was used to double enter and clean the data. Data analyses were performed using SPSS 17.0™ (SPSS Inc., Chicago, IL, USA). Although the nature of recruitment model for this study ultimately did not allow us to calculate STI prevalence rates for this population, we did collect and analyze data on participants' risk behaviors that previous studies have indicated were associated with STI infection.

### Ethics

Trained interviewers explained the purpose, process, benefits and potential risks before obtaining written informed consent from participants. Study participation was both anonymous and voluntary. The study was approved by the institutional review board of the Center for Disease Control and Prevention, Chaoyang District, Beijing, China.

## Results

### Sociodemographic characteristics

Of 224 women, 89.7% were ethnic Han and 33.9% were local residents of Beijing. Their average age was 25.6 years (SD±4.6; range 19–46 years) and 93.7% had a university-level education. Homosexual self-identification was cited by 67.4% (151/224). Students represented 32.6% (73/224) of participants. The 37 couples represented one-third (74/224) of the total subjects. Sociodemographic characteristics comparing women who only had sex with women and women who had sex with both women and men in the past year were similar, except for sexual orientation (Table 1).

**Table 1:** Sociodemographic characteristics of women who have sex with women, Beijing, China, 2010–2011.

Characteristics	No.(%) of WSW total n = 224	No.(%) of WSW/M in the past year^1^ n = 24	No.(%) of WSW exclusive in the past year^1^ n = 200
***Age (years)***			
**18–19**	6(2.7)	0(0)	6(3.0)
**20–29**	188(83.9)	20(83.3)	168(84.0)
**30–39**	26(11.6)	3(12.5)	23(11.5)
**40–49**	4(1.8)	1(4.2)	3(3.0)
***Marital status***			
**Unmarried, living with female partner**	95(42.4)	7(29.2)	88(44.0)
**Unmarried, living alone or with parents**	116(51.8)	14(58.3)	102(51.0)
**Married, living with male spouse**	4(1.8)	1(4.2)	3(1.5)
**Other** 2	9(4.0)	2(8.3)	7(3.5)
***Education***			
**Junior high school or below**	4(1.8)	0(0)	4(2.0)
**Senior high school**	10(4.5)	0(0)	10(5.0)
**Undergraduate**	177(79.0)	21(87.5)	156(78.0)
**Graduate and above**	33(14.7)	3(12.5)	30(15.0)
***Income (US$/month)***			
**None**	69(30.8)	5(20.8)	64(32.0)
**Up to $311**	20(8.9)	3(12.5)	17 (8.5)
**>$311–466**	41(18.3)	6(25.0)	35(17.5)
**>$466–622**	36(16.1)	6(25.0)	30(15.0)
**>$622**	58(25.9)	4(16.7)	54(27.0)
***Beijing local resident***			
**Yes**	76(33.9)	11(45.8)	65(32.5)
**No**	148(66.1)	13(54.2)	135(67.5)
***Self-reported sexual orientation***			
**Homosexual**	151(67.4)	4(16.7)	147(73.5)
**Heterosexual**	1(0.4)	1(4.2)	0(0)
**Bisexual**	61(27.2)	16(66.7)	45(22.5)
**Undecided**	11(4.9)	3(12.5)	8(4.0)

1 “WSW/M in the past year” refers to women who had had sex with both women and men in the past year, and “WSW exclusive in the past year” refers to women who had only had sex with women in the past year.

2 Other: 1 participant was married but living alone; 5 were divorced/widowed living alone; 2 were living with a man (not a spouse); 1 was living with a woman (not a partner).

### Sexual behaviors

The average age of sexual initiation was 20.5 years; 74.1% of participants reported that their first sexual experience was with a female. The average age of sexual initiation with a same-sex partner was 21.5 years.

During the past year, 34.3% (77/224) reported a history of >1 sexual partner and 72.4% (162/224) had ever had >1 sexual partner. In the year preceding their participation in the study, 92% (206/224) of women reported sexual relations with women, 26.8% (60/224) of whom had had sex with two women or more. Sexual relations with men in the past year were reported by 10.7% (24/224) of participants. Condom use in the last sex with a man was reported by 54.2% (13/24) of women; 12.5% (3/24) reported never having used a condom with a man in the past year. The consistent use of condoms in sexual relations with women over the past year was reported by 11.1% (23/208).

No one reported exchanging sex for money or goods. Only 3.6% (8/224) of participants said they had ever used drugs, 50% (4/8) of whom reported having had sex after taking drugs. All eight women reported using ‘club drugs’ such as ketamine and Ecstasy. No one reported injecting drugs.

Among the participants, 79.5% (178/224) reported drinking alcohol, 46.6% (83/178) of whom reported drinking before engaging in sex. Of the 178 women who reported drinking, 0.6% (1/178), reported drinking at least once per day, 29.2% (52/178) at least once per week, 50% (89/178) at least once per month, and 20.2% (36/178) at least once per year. A total of 34.8% (62/178) participants reported on average drinking less than 1 drink ( = 50 g), 30.9% (55/178) 1∼2 drinks, 13.0% (23/178) 2∼3 drinks, and 21.3% (38/178) >3 drinks per drinking event.

Sexual behaviors with female partners were highly variable. Digital and/or oral clitoral/vaginal contacts were most common (Table 2). In the past year, 13.4% (30/224) reported using sex toys with their female partners; of these, 43.3% (13/30) reported consistent condom use with the sex toys and 36.7% (11/30) had shared sex toys. There was high awareness of the so-called “G-spot”; 65.2% (120/184) of women reported that their “G-spot” had been stimulated during sex, 49.2% (59/120) of whom reported bleeding during or after sex. Only 12.5% (8/64) of those never reporting “G spot” stimulation reported bleeding during or after sex (P<0.001).

**Table 2:** Specific sexual behaviors of women who have sex with women in Beijing, China.

Sexual activities	No. (n = 224)	%
**Digital-clitoral contact**	205	91.5
**Digital-vaginal contact**	202	90.2
**Oral-clitoral contact**	165	73.7
**Oral-vaginal contact**	131	58.5
**Vaginal-vaginal contact**	89	39.7
**Use sex toys with clitoris**	37	16.5
**Use sex toys with vagina**	33	14.7
**Digital-anal intercourse**	3	1.3

## Discussion

This is the first reported study in China to explore the behavioral factors of WSW that have been associated in other studies with the transmission of STI/HIV. It also represents one of the largest WSW behavioral surveys ever published. The strong association between seeking the G-spot and bleeding during sex was a surprise and suggests a serious issue for discussion when addressing both sex during menstruation and “rough” sex. Sexual activities were varied and sometimes medically traumatic.

Currently there is a dearth of research that has examined the specific factors related to infection [38]. Because of the recruitment model we were unable to determine whether having sex with men [6], injection drug use [37], multiple bisexual partners [43] and other factors previously reported as STI/HIV risk factors among WSW were significant in this study, but as WSW in our study were unlikely to have had sex with a man in the past year (10.7%), we surmise that risk of woman-to-woman STI and RTI spread might have been underestimated in previous studies.

Prior studies have suggested that oral sex [44], vaginal penetration with fingers, and mutual masturbation were the most commonly reported sexual practices between women [19], [43]. Some WSW in our study reported that vaginal-vaginal contact with their female partners made them feel much closer emotionally due to the full body contact, compared with other sexual activities. More than one third of the participants in this study reported vaginal-vaginal sex behavior, and of these, 40.2% had experienced bleeding during sex. Vaginal-vaginal contact can cause a direct exchange of secretions and is the presumed risk mode of transmission for WSW, though use of toys, traumatic sex, and sex with men cannot be ruled out.

The data showed that sexual behaviors between women were similar to those between women and men, with the main difference being digital vs. penile vaginal penetration. STI/AIDS research and interventions focus predominantly on penile penetration sex; a broader scope of focus is indicated for work and research with WSW.

Seeking the G-spot might be relevant to evaluating risk in Chinese WSW. Because HIV/STI infections can spread through vaginal secretions and menstrual blood [45], trauma from G-spot exploration may increase risk of STI by disrupting the integrity of the genital mucosa. Sexual health education should highlight the merits of gentle sexual exploration, and the benefits of avoiding trauma and bleeding. We found that during post-survey discussions, some WSW participants mentioned that compared to penis-vaginal intercourse, digital-vaginal intercourse is harder and more excessive forceful, and consequently constitutes ‘rough sex’ that may cause bleeding. Because rough sex in digital-vaginal intercourse can happen with both WSW and WSM, it is possible that avoiding this particular behavior could reduce the possibility of STI transmission.

Bleeding during or after sex may increase the susceptibility of mucous membrane exposure to infectious agents through bleeding, or may indicate that higher risk partners may practice sexual behaviors that involve bleeding (menstruation and/or physical trauma). It is also plausible that women having more frequent sexual activity might report sex and bleeding more often (related to menstruation, for example), such that frequency of sex is confounded with bleeding. In order to promote STI prevention in WSW as well as in the general female population, avoiding sexual activity that causes bleeding should be stressed. If a woman bleeds as a result of sexual activity she should be encouraged to seek medical care.

Most of our WSW participants were aged from 20 to 29 years old, similar to the sample populations of other WSW studies [11]. There is relatively low social pressure for urban women to get married in their twenties in China. Young WSW may also have more exploratory attitudes toward same-sex behavior. In addition, many of the sample population were students who live in a comparatively freer social atmosphere than young women who are employed. Our participants had high levels of education (93.7% with a college education) and were relatively young, similar to populations in other studies [19], [21]. This might imply that these women would have had a relatively low risk of acquiring STIs, but this was contrary to our findings regarding their risk behaviors.

Most of participants were WSW without Beijing local residency. We speculate that non-residents might face difficulties in maintaining long-term relationships due to economic instability and the lack of a private dwelling. Beijing-resident unmarried WSW typically lived with their parents, which might limit their chances to have sex with others. While drug use was uncommon, 79.5% (178/224) of participants reported taking alcohol, which is also a potential STI risk behavior [46], [47].

This study demonstrates the need for medical evaluations to consider WSW issues, nearly always overlooked in China [48]. That 25.9% of WSW participants reported that their first sexual experiences were with men and 10.7% maintained sexual relations with men during the past year are consistent with a UK study that also found a proportion of WSW reporting sexual activity with men in the past year [49]. The fact that a woman is practicing homosexual behaviors at present does not mean that these are the only sexual behaviors she has practiced or will practice. Furthermore, although some WSW in the study had never had male sexual partners, their female partners may previously have had sex with men. Some women reported unprovoked during the interviews that during their annual health appointments, doctors refused to perform gynecological/STI examinations if the women indicated they had not had sex with men; sex with women was almost never broached. Women felt inhibited discussing their sexual orientations and related sexual health problems with both male and female physicians, but reported feeling more so with male physicians.

Strengths of our study include its comprehensive assessment of detailed risk profiles for STI/RTI. The study was announced through a variety of venues, including lesbian bars, salons, and nongovernmental organizations, but most participants reported learning about it through the internet. This strength is also a potential limitation in that it may have induced a selection bias in the sample. Approximately one-third of participants were couples and this may also have induced a selection bias in the sample, but the data were still considered reliable because the survey sought information on comprehensive sexual history, not only history with the most recent partner. It is also difficult to determine the exact recruitment source of each participant because multiple recruitment methods were used. For instance, some women were referred by their friends to the study, after which they read the blog to learn the details, and then came to volunteer. The proportion of the population with access to the internet most likely has an above-average income and education level, which may not be representative of the general population. Social desirability bias may also have been an issue in this study due to the sensitivity of the questions on sexual identity, sexual behavior [49], and drug use. Although all women received STI screening, a limitation of the study is that suboptimal diagnostic tests were used with low sensitivity (data not presented).

At present, consciousness of WSW sexual health is dismally low in China, as in much of the rest of the world. Given that Beijing WSW have sexual practices that put them at considerable risk of STI/RTI, screening, recognition, intervention, and prevention of adverse sexual, reproductive, and general health outcomes should be considered priorities for this population.
